# Covalent organic framework with sulfonic acid functional groups for visible light-driven CO_2_ reduction†

**DOI:** 10.1039/d2ra02660k

**Published:** 2022-06-17

**Authors:** Wanrong Li, Qian Wang, Fuzhi Cui, Guofang Jiang

**Affiliations:** College of Chemistry and Chemical Engineering, State Key Laboratory of Chemo/Biosensing and Chemometrics, Advanced Catalytic Engineering Research Center of the Ministry of Education, Hunan University Changsha 410082 P. R. China guofangjiang@hnu.edu.cn

## Abstract

In this study, a covalent organic framework (TpPa–SO_3_H) photocatalyst with sulfonic acid function groups was synthesized using a solvothermal method. The morphologies and structural properties of the as-prepared composites were characterized by X-ray diffraction, infrared spectroscopy, ultraviolet-visible diffuse reflectance spectroscopy, X-ray photoelectron spectroscopy, N_2_ adsorption–desorption measurements, and field emission scanning electron microscopy. An electrochemical workstation was used to test the photoelectric performance of the materials. The results show that TpPa–SO_3_H has –SO_3_H functional groups and high photocatalytic performance for CO_2_ reduction. After 4 h of visible-light irradiation, the amount of CO produced is 416.61 μmol g^−1^. In addition, the TpPa–SO_3_H photocatalyst exhibited chemical stability and reusability. After two testing cycles under visible light irradiation, the amount of CO produced decreased slightly to 415.23 and 409.15 μmol g^−1^. The XRD spectra of TpPa–SO_3_H were consistent before and after the cycles. Therefore, TpPa–SO_3_H exhibited good photocatalytic activity. This is because the introduction of –SO_3_H narrows the bandgap of TpPa–SO_3_H, which enhances the visible light response range and greatly promotes the separation of photogenerated electrons.

## Abstract 2

In this study, covalent organic framework (TpPa–SO_3_H) photocatalyst with sulfonic acid function groups was synthesized using a solvothermal method.

## Graphical abstract

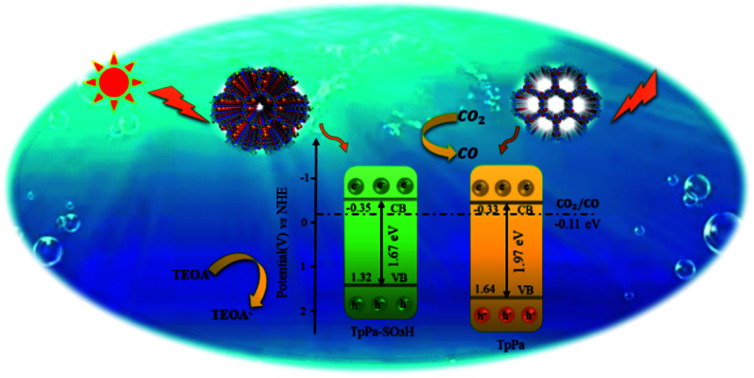

## Introduction

Rapid industrial development is associated with the combustion of fossil fuels. However, fossil fuels are a limited resource, and dependence on them will result in an energy crisis. Moreover, burning fossil fuels increases the concentration of CO_2_ in the atmosphere. CO_2_ is one of the main greenhouse gases responsible for global warming, which could affect the development and survival of human beings.^1,2^ As environmental issues attract increasing attention worldwide, the utilization of resources and the reduction of CO_2_ emissions have become important considerations. Therefore, it is imperative to develop clean and green CO_2_ conversion technology.^3,4^

Semiconductor photocatalysis technology can transform solar energy into chemical energy without pollution, and they are considered to be the most effective means of alleviating the energy crisis and environmental pollution.^5,6^ As early as 1979, Fujishima used photocatalytic technology to reduce CO_2_,^7^ and there have since been many reports on photocatalysts. Common CO_2_ reduction photocatalysts include inorganic semiconductors,^8–10^ metal–organic frameworks (MOFs),^11^ and covalent organic frameworks (COFs).^12^ In particular, TiO_2_ is widely used for photocatalytic CO_2_ reduction because of its low cost, high stability, and low toxicity.^13^ However, it has a wide band gap (3.2 eV) so efficient photocatalytic performance cannot be achieved with visible light. Therefore, it is necessary to use physical or chemical methods to modify TiO_2_ and improve its photocatalytic efficiency.^14^ In contrast, g-C_3_N_4_ has a narrow band gap (2.7 eV), which results in high photocatalytic activity and an excellent visible light response.^15^ However, owing to the short recombination lifetime of fast charge carriers, the charge separation of g-C_3_N_4_ is insufficient. Moreover, it has low crystallinity and a small specific surface area, so it cannot make full use of light, and its photocatalytic performance is limited.^16^ MOFs can effectively capture light, shorten the carrier transmission distance, and enhance the separation of electrons and holes, so they can be used as new functional materials for photocatalysts.^17,18^ However, MOFs are not functionalized or synthesized with heterojunction materials, and they do not have high photocatalytic activity and stability. Therefore, it is often necessary to modify MOFs to improve their photocatalytic performance.^19^ Conjugated microporous polymers (CMPs) can capture light and have an effective charge separation capability, so they have been extensively studied in the field of photocatalysis.^20–22^ However, the formation of CMPs is controlled by kinetics, and they are linked by irreversible organic covalent bonds. Therefore, the structure is amorphous and forms a disordered microporous polymer.^23^

COFs are new two- or three-dimensional organic crystalline polymer materials composed of light elements (*e.g.*, C, N, O, and B), which have adjustable pore diameters, low density, and strong stability.^18,24^ Moreover, they have excellent semiconductor characteristics for photocatalysis, such as good absorption of visible light, a suitable band gap, and fast charge carrier mobility.^25,26^ COFs have π–π conjugated units, which can maintain good chemical stability under acidic and alkaline conditions and in different organic solvents.^27,28^ Furthermore, COFs have a large specific surface area, which exposes more photocatalytic sites, thereby increasing the light absorption capacity. Owing to their excellent characteristics, COFs are widely used as efficient photocatalytic catalysts for CO_2_ reduction,^29^ degradation of organic pollutants,^30^ Cr(vi) reduction,^31^ water decomposition,^32^ hydrogen evolution,^33,34^*etc.*

**Fig. 1:**
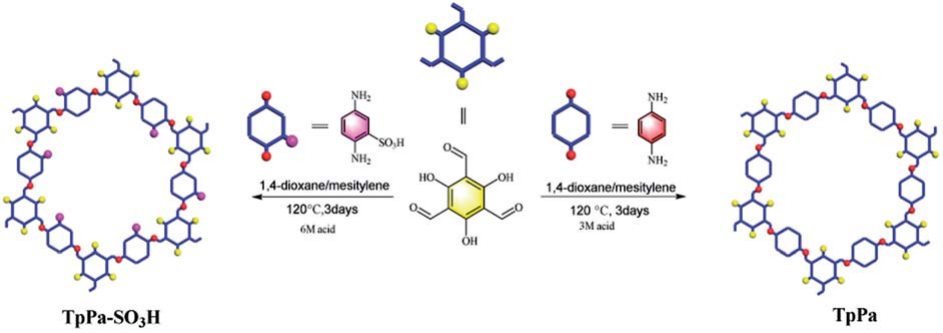
Schematic diagram of synthesis of the TpPa–SO_3_H and TpPa photocatalysts.

Herein, we report the covalent organic framework TpPa–SO_3_H with a sulfonic acid function groups for the photocatalytic reduction of CO_2_ (Fig. 1). Compared to TpPa, TpPa–SO_3_H is expected to exhibit excellent light absorption ability and abundant photocatalytic activity sites under visible-light illumination. The introduction of –SO_3_H group can reduce the band gap, it will effectively promote the transfer and separation of interface charges, accelerate the migration of carriers, and inhibit the recombination of electrons and holes. Thus, TpPa–SO_3_H will have great potential in visible light-driven reduction of CO_2_ emissions.

## Experimental

### Synthesis and characterization

**Fig. 2:**
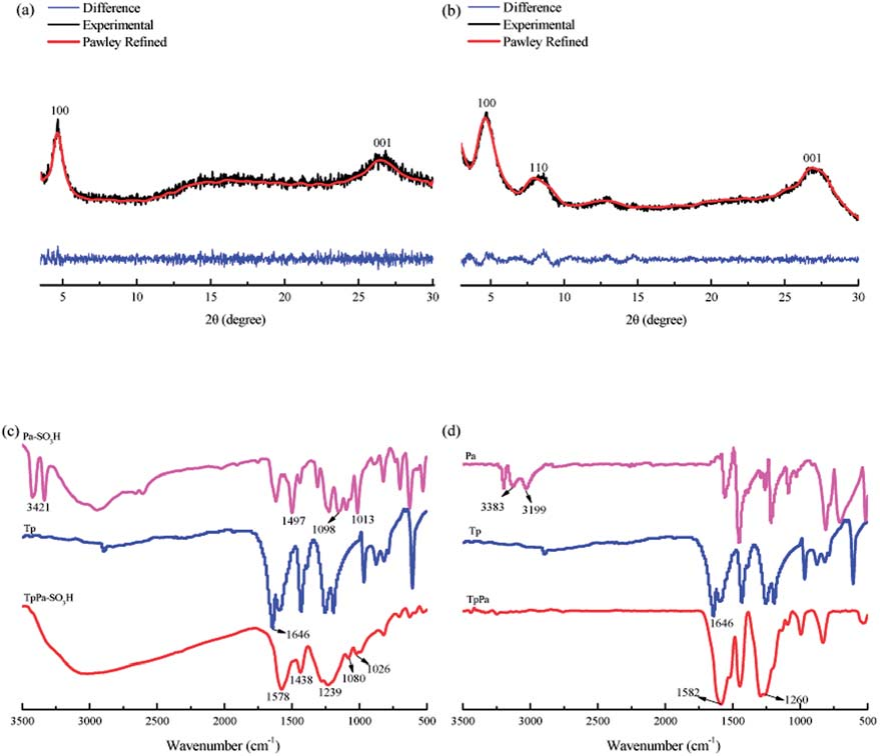
XRD patterns of (a) TpPa–SO_3_H and (b) TpPa. FT-IR spectra of (c) TpPa–SO_3_H and (d) TpPa.

**Fig. 3:**
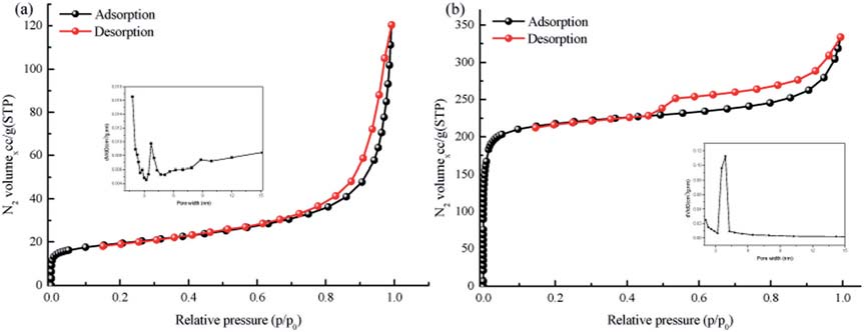
N_2_ adsorption–desorption isotherms of (a) TpPa–SO_3_H and (b) TpPa.

**Fig. 4:**
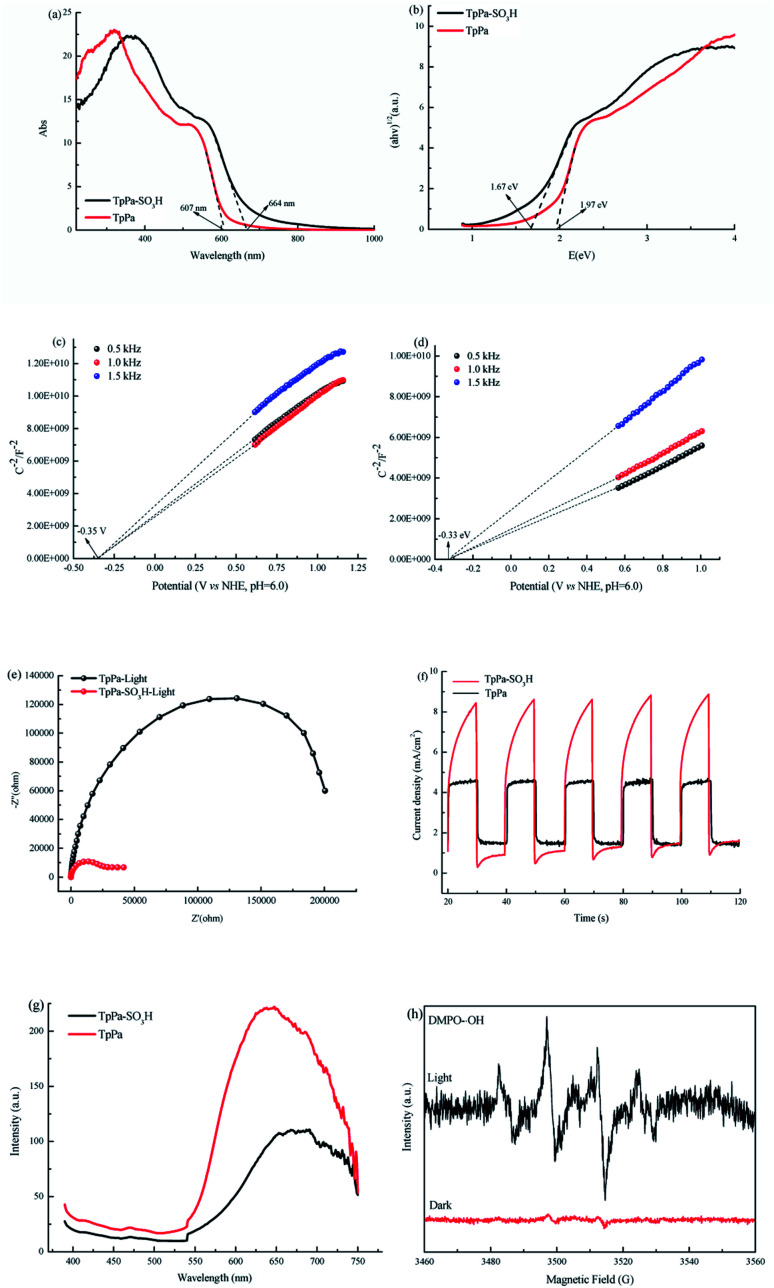
UV-vis DRS images of (a) TpPa–SO_3_H and TpPa. (b) Plots of (*αhν*)^1/2^ as a function of energy (*hν*) for bandgap energies of TpPa–SO_3_H and TpPa. Mott–Schottky (MS) plots of (c) TpPa–SO_3_H and (d) TpPa. (e) Nyquist plot of electrochemical impedance spectra (EIS) of TpPa–SO_3_H and TpPa. (f)Transient photocurrent response of TpPa–SO_3_H and TpPa. (g) Photoluminescence (PL) emission spectra of TpPa–SO_3_H and TpPa. (h) Electron spin resonance (ESR) spectra of DMPO–˙OH under visible light irradiation.

The TpPa–SO_3_H and TpPa were synthesized according to a solvothermal procedure. We synthesized two kinds of imine-linked COFs through the reaction of 2,4,6-trihydroxy-1,3,5-benzenetricarbaldehyde (Tp) with two different monomers 2,5-diaminobenzenesulfonic acid (Pa–SO_3_H) and *p*-phenylenediamine (Pa) (Fig. S1 and S2†). Thermogravimetric analysis (TGA) showed that TpPa–SO_3_H and TpPa had similar thermogravimetric curves. Compared to TpPa–SO_3_H, TpPa showed higher thermal stability, but both materials exhibited good thermal stability at room temperature (∼25 °C) and pressure (Fig. S3†). X-ray diffraction (XRD) was used to determine the crystal structural of TpPa–SO_3_H and TpPa (Fig. 2a and b). The XRD pattern of TpPa–SO_3_H showed two main characteristic peaks at 2*θ* = 4.67° and 26.62°, which correspond to the 100 and 001 planes, respectively. TpPa showed a main diffraction peak at 4.79°, which corresponds to the 100 plane. There was a slightly wider diffraction peak at 8.54°, which can be attributed to the 110 plane. Finally, the diffraction peak at 27.04° is assigned to the 001 plane. The peak positions of the synthesized COFs were consistent with the simulated positions, indicating that TpPa–SO_3_H and TpPa were successfully synthesized. The structures of TpPa–SO_3_H and TpPa were investigated using FT-IR (Fig. 2c and d). Fig. 2c shows that Tp had a stretching vibration absorption peak associated with C=O at 1646 cm^−1^, and Pa–SO_3_H had a telescopic vibration associated with –NH_2_ at 3421 cm^−1^. There were also symmetric and asymmetric stretching bands associated with O=S=O at 1013, 1098, and 1497 cm^−1^, indicating the presence of sulfonic acid groups. The stretching bands at 1646 cm^−1^ for C=O and 3421 cm^−1^ for –NH_2_ disappeared in the synthesized products. Symmetric and asymmetric stretching bands were observed at 1578 cm^−1^ for C=C and 1239 cm^−1^ for C–N, and 1026, 1080, and 1438 cm^−1^ for O=S=O. This indicates that TpPa–SO_3_H was synthesized successfully. The Fourier transform infrared spectrum of TpPa showed that the C=O stretching band of Tp at 1643 cm^−1^ and the stretching vibration bands of the NH_2_ group of Pa at 3383 and 3199 cm^−1^ disappeared, indicating that the monomers were completely consumed. Moreover, new C=C and C–N stretching vibration bands appeared at 1582 and 1260 cm^−1^, respectively. Thus, TpPa was synthesized successfully. Full-spectrum and energy dispersive X-ray (EDX) elemental mapping showed that TpPa–SO_3_H is composed of four elements: C, N, O, and S (Fig. S4 and S6a†). The C 1s spectrum was convolved into three peaks with binding energies of 284.8, 286.16, and 289.64 eV, corresponding to C–C, C=N or C–O, and C=O bonds, respectively. The high-resolution XPS spectrum of O 1s deconvolved into two peaks with binding energies of 531.5 and 532.85 eV corresponding to C–O and C=O bonds, respectively. The high-resolution X-ray photoelectron spectroscopy (XPS) spectrum of S 2p. One binding energy is attributed to S 2p_3/2_ at 168.1 eV, and the other to S 2p_1/2_ at 169.3 eV. This confirms that the TpPa–SO_3_H structure was formed (Fig. S4†). Similarly, TpPa is composed of C, N, and O elements (Fig. S5 and S6†). C 1s was convolved into three peaks with binding energies of 284.8, 285.93, and 289.25 eV, corresponding to C–C, C=N or C–O, and C=O bonds, respectively. The high-resolution XPS spectrum of O 1s deconvolved into two peaks with binding energies 530.98 and 532.76 eV corresponding to C–O and C=O bonds, respectively (Fig. S5†). Within the relative pressure range *P*/*P*_0_ < 0.1, the proportion of TpPa–SO_3_H and TpPa increased sharply. This may be due to some structural condensation in the synthesis process. According to the IUPAC classification, under relative pressure, both materials have an H3 hysteresis loop, and the isotherms are similar to type IV isotherms. Therefore, owing to the existence of mesopores. The Brunauer–Emmett–Teller (BET) specific surface areas of TpPa–SO_3_H and TpPa were 63.61 and 779.62 m^2^ g^−1^, respectively. The BET specific surface area of TpPa was higher than that of TpPa–SO_3_H, possibly owing to the introduction of the –SO_3_H group to the benzene ring. The pore size of TpPa–SO_3_H is smaller than that of TpPa owing to the introduction of the –SO_3_H group. The pore sizes of TpPa–SO_3_H and TpPa are 3.68 and 4.28 nm, respectively, and the pore volumes are 1.22 and 0.52 cm^3^ g^−1^, respectively (Fig. 3). The maximum CO_2_ adsorption capacities of TpPa–SO_3_H and TpPa were 34.28 and 57.47 cm^3^ g^−1^ (Fig. S7†), respectively. The adsorption enthalpy Δ*H* is the isosteric heat of adsorption *Q* produced during the adsorption process. This is calculated using the Clausius–Clapeyron equation, ln *P* = −Δ*H*/*RT* + *C*. Therefore, the CO_2_ isosteric heat of adsorptions *Q* of TpPa–SO_3_H and TpPa are −34.08 and −33.61 kJ mol^−1^, respectively. Scanning electron microscopy (SEM) and transmission electron microscopy (TEM) images of the TpPa–SO_3_H and TpPa show that the surface of TpPa is smooth and flat with a clustered structure, and the introduction of –SO_3_H makes the surface rough, with an obvious fibrous structure. Ordered structures can provide efficient transport paths for reactants, and more active sites for the photocatalytic reactions (Fig. S8†). UV-vis diffuse reflectance spectroscopy (DRS) was used to study the light absorption and photochemical properties of the two materials. The semiconductor bandgap energy *E*_g_ was calculated using the Tauc plot method. The bandgap energy *E*_g_ of a semiconductor can be calculated using the equation (*αhν*)^1/*n*^ = *A*(*hν* − *E*_g_), where *α* is the optical absorption coefficient, *h* is the photon energy, *ν* is the frequency, *E*_g_ is the forbidden bandwidth of the material, *A* is a proportional constant, and the *n* indicates the type of optical transition of the semiconductor. Here, the value of *n* is 2, therefore, both materials are indirect bandgap semiconductors. The light absorption range of TpPa–SO_3_H is greater than that of TpPa. Thus, according to the Tauc mapping method, the electron energies of TpPa–SO_3_H and TpPa are 1.61 and 1.97 eV, respectively. The bandgap of TpPa–SO_3_H was narrower than that of TpPa. Therefore, TpPa–SO_3_H has stronger visible-light absorption capability (Fig. 4a and b). The photoelectric properties of TpPa–SO_3_H and TpPa were tested using a CHI660e three-stage electrochemical workstation, and the carrier separation efficiency was investigated further. Mott–Schottky (MS) curves with frequencies of 500, 1000, and 1500 Hz were used under dark conditions (Fig. 4c and d). The MS method was used to analyze the conduction bands of TpPa–SO_3_H and TpPa. That is, a straight line was drawn from the MS curve to the *x*-axis to obtain the conduction band potentials, which were −0.35 and −0.33 V for TpPa–SO_3_H and TpPa, respectively. Fig. 4c and d shows that the slopes of the MS curves for TpPa–SO_3_H and TpPa were positive, which confirms that both materials are n-type semiconductors. Electrochemical impedance spectroscopy (EIS) and transient photocurrent (PC) response measurements were used to further study the carrier separation efficiency. Under illumination, Nyquist semicircle radius of TpPa–SO_3_H was much smaller than that of TpPa (Fig. 4e). This shows that the introduction of –SO_3_H can improve the charge-transfer ability of the material. To evaluate the charge separation abilities of TpPa–SO_3_H and TpPa, the switching period photocurrent responses of the two materials under light irradiation were measured (Fig. 4f). The photocurrent distribution is shown in Fig. 4f, TpPa–SO_3_H produced a stronger photocurrent than TpPa. This shows that the introduction of the –SO_3_H group can effectively promote charge separation and accelerate the separation of charge carriers. Compared to TpPa, TpPa–SO_3_H showed a lower PL intensity (Fig. 4g). The PL decay profiles, fitting parameters and average lifetimes (Fig. S9 and Table S1†) reveal that the PL lifetime (*τ*) of TpPa–SO_3_H (0.5 ns) is shorter than those of TpPa (0.7 ns), the results show that the recombination rate of e^−^–h^+^ in TpPa was very high, and the introduction of the SO_3_H group provided more effective e^−^–h^+^ separation.

### Photocatalytic reduction of CO_2_

**Fig. 5:**
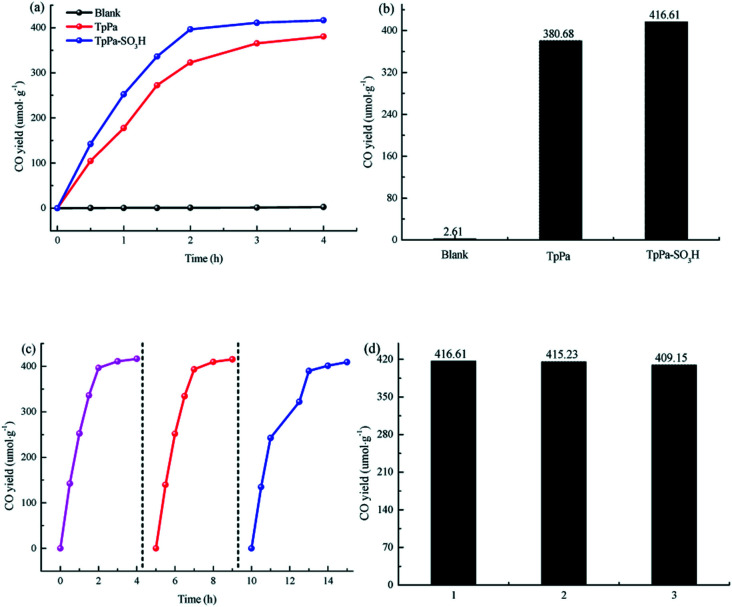
(a and b) Photocatalytic activity of the samples. (c and d) Repetitive testing of the CO_2_ photoreduction process with TpPa–SO_3_H under simulated solar light irradiation.

**Fig. 6:**
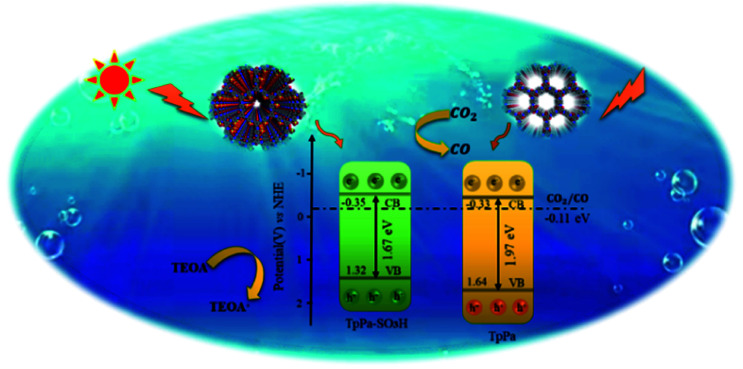
Schematic illustration of the photocatalysis process of TpPa–SO_3_H and TpPa.

The photocatalytic activities of TpPa–SO_3_H and TpPa were evaluated based on the photocatalytic reduction of CO_2_ under simulated visible light (Fig. 5a and b). In the absence of CO_2_, the other conditions remained unchanged. COFs do not produce CO when they decompose, so this can be excluded from consideration. Fig. 5a and b show that without the photocatalysts, the photocatalytic activity of CO_2_ was very low, and after 4 h of visible light irradiation only 2.61 μmol g^−1^ of CO was produced. The addition of a photocatalyst was the main factor affecting the reduction of CO_2_. After 4 h of simulated sunlight irradiation, the CO yields with TpPa–SO_3_H and TpPa were 416.61 and 380.68 μmol g^−1^, respectively. After 2 h of illumination, there are almost no increase of CO amount over TpPa–SO_3_H, the reason may be that the amount of photocatalyst is low and the active sites are insufficient. This shows that the photoreduction ability of CO_2_ can be improved by adding –SO_3_H substituents to the monomers of synthetic materials. To demonstrate the high selectivity and stability of the photocatalyst materials under visible light, the materials were recovered and subjected to cyclic testing (Fig. 5c and d). The photodegradation efficiency of TpPa–SO_3_H did not change significantly. After two reaction cycles, an excellent constant CO yield was obtained. This indicates that TpPa–SO_3_H has good photocatalytic stability. To further prove the stability of the TpPa–SO_3_H photocatalytic material, XRD was used to determine whether the crystallinity had changed (Fig. S10†). There were no obvious differences in the XRD patterns before and after the reaction, indicating that the material had good structural stability. Next, the energy-band structure of the material was calculated (Fig. 6). Under visible-light illumination, the CB potentials of TpPa–SO_3_H and TpPa had larger negative values than those of the CO_2_/CO redox potential. Thus, the photocatalytic activity for CO_2_ conversion was higher, and the thermodynamic requirements for CO_2_/CO conversion were satisfied. Electron spin resonance (ESR) analysis was conducted to identify free radicals. After testing, no ˙O_2_^−^ signals were observed, but the ˙OH signal was observed (Fig. 4h). Therefore, we propose a possible mechanism for this process. TpPa–SO_3_H is photoexcited to the conduction band by electrons (e^−^) in the valence band, which generates holes (h^+^) in the valence band. Then, e^−^ and h^+^ can be transferred to the surface of the catalyst, where h^+^ oxidizes H_2_O into ˙OH and H ions (H^+^). Ru(bpy)_3_Cl_2_ was introduced as a photosensitizer to capture light and improve the efficiency of the photocatalytic reduction of CO_2_. [Ru(bpy)_3_]^2+^ can effectively receive photogenerated e^−^ and H^+^ reduces CO_2_ to CO. Meanwhile, TEOA was introduced as a sacrificial agent to prevent the recombination of photogenerated e^−^ and h^+^. The h^+^ in the valence band easily accepts the e^−^ from TEOA, which is then oxidized to TEOA^+^. This can be summarized by the equations:TpPa–SO_3_H + *hν* → e^−^ + h^+^h^+^ + H_2_O → ˙OH + H^+^2H^+^ + CO_2_ + 2e^−^ → CO + 2H_2_O.

The photocatalytic reduction efficiencies of TpPa–SO_3_H and TpPa under visible-light irradiation were compared with various other photocatalysts (Table S2†). Compared to most photocatalytic materials, TpPa–SO_3_H has greater photocatalytic activity, and a higher photocatalytic CO production rate. The results show that the COFs synthesized by introducing –SO_3_H group into the monomer had a positive effect on the photocatalytic activity for CO_2_ reduction.

## Conclusions

In summary, 2D covalent organic framework TpPa–SO_3_H and TpPa photocatalysts, were synthesized and applied to visible light-driven CO_2_ reduction. The results show that the introduction of sulfonic acid function groups gives TpPa–SO_3_H high photocatalytic activity for CO_2_ reduction. TpPa–SO_3_H showed excellent light absorption ability and abundant photocatalytic activity under visible light. Moreover, it exhibited a higher yield and greater stability than TpPa. After 4 h of visible light irradiation, TEOA was introduced as a sacrificial agent, and [Ru(bpy)_3_]Cl_2_·6H_2_O was used as photosensitizer, the photocatalytic reduction of CO_2_ by TpPa–SO_3_H produced CO with a yield of 416.61 μmol g^−1^. The –SO_3_H group can affect the catalytic activity, it can promote H_2_O to produce more H^+^. Under light irradiation, synergistic effect of H^+^ and e^−^ promoted the reduction of CO_2_ to CO. This study provides a method for the design of COFs photocatalysts. In addition, it can be used to effectively reduce environmental pollution and alleviate the energy crisis.

## Conflicts of interest

There are no conflicts to declare.

## Supplementary Material

RA-012-D2RA02660K-s001
