# Supplementary material for: Covalent organic framework with sulfonic acid functional groups for visible light-driven CO2 reduction
Source: RSC Adv. 2022 Jun 17;12(28):17984–9. doi: 10.1039/d2ra02660k (PMC9204709; doi:10.1039/d2ra02660k)
Supplement: RA-012-D2RA02660K-s001 [file RA-012-D2RA02660K-s001.pdf]

Electronic Supplementary Material (ESI) for RSC Advances.
This journal is © The Royal Society of Chemistry 2022

Covalent organic framework with sulfonic acid functional groups for visible light-driven CO_2 reduction

Wanrong Li, Qian Wang, Fuzhi Cui*, Guofang Jiang*,

Advanced Catalytic Engineer Research Center of the Ministry of Education, College of Chemistry and Chemical Engineering, Hunan University, Changsha 410082, China.

Materials Characterization

The crystal structure of the material was analyzed by X-ray powder diffractometer(XRD, Bruker D8 Advance). Fourier transform infrared spectrometer(FT-IR, TENSOR 27) was used to analyze the composition and structure of photocatalyst. The elemental composition and electronic structure of the materials were analyzed by X-ray photoelectron spectroscopy (XPS, AXIS SUPPA). Surface area of the catalysts used to analyze by N_2 adsorption and desorption isotherms with automatic pore size analyzer Brunauer-Emmett-Teller (BET, JW-BK 200C). The thermal stability of the material was tested by the thermogravimetric-differential thermal synchronization analyzer (TGA, STA 7200). Using automatic pore size analyzer (BET Micromeritics ASAP) to study the amount of adsorption of CO_2. Field emission scanning electron microscope (SEM, Hitachi) was used to observe the surface morphology of the material. The ordered structure of the materials were observed by high resolution transmission electron microscope(HRTEM,Titan G260-300). Ultraviolet integrating sphere (DRS, UV-2600) were used to test the photoelectric properties of the materials, test the background baseline with $BaSO_4$ powder, calculation of band gap energy E_g of semiconductor by Tauc plot method. Electron spin

resonance (ESR, Bruker EMXplus-6/1) was used to measure the key active substances. Use a high-performance fluorescence spectrometer(PL, FP-8500) and time-resolved fluorescence (TRPL, Edinburgh FLS1000) to further illustrate the charge separation role of materials. CHI660e three-stage electrochemical workstation is used to further study the separation efficiency of charge carriers. Under the irradiation of xenon lamp, the photocurrent transient response (PC), electrochemical impedance (EIS) and Mott-Schottky curves (MS) at 500, 1000 and 1500 Hz in the switching period were measured respectively. Platinum sheet was used as counter electrode, Ag/AgCl electrode was used as the reference electrode, and tin dioxide glass (FTO) coated with 1×1 cm^2 material was used as working electrode, 0.5 M Na_2SO_4 aqueous solution was used as the electrolyte.

Synthesis of TpPa-SO_3H and TpPa

TpPa-SO_3H and TpPa were synthesized by solvothermal method. Tp (63 mg, 0.3 mmol) and Pa-SO_3H (84.7 mg, 0.45 mmol) /Pa (48.6 mg, 0.45 mmol) were dissolved in a mixture of solvents of 1,4-dioxane/mesitylene (V:V = 1/4) and ultrasonicated for 15 min. Then, 0.3 ml of 6 M acetic acetic acid aqueous solution was added. The ampoule bottle was flash frozen in liquid nitrogen, The mixture was heated at 120°C for 3 days in an oven.The cooled dark red and red products were collected by centrifugation and washed three times with 1,4-dioxane, water and acetone, respectively. and finally dried at 120°C in a vacuum oven for 8 h.[1,2]

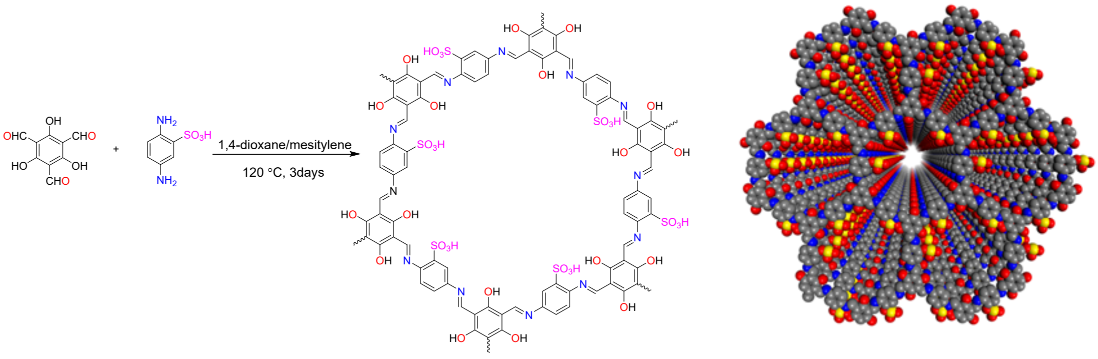

Fig.S1. Synthesis schematic of TpPa-SO_3H.

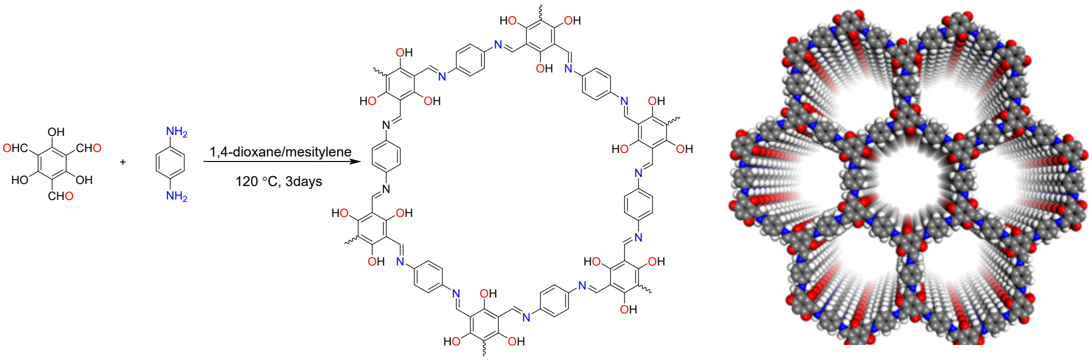

Fig.S2. Synthesis schematic of TpPa.

Photocatalytic CO_2 reduction experiments

Photocatalytic reduction of CO_2 with Xe lamp as light source. The all-glass automatic online trace gas analysis system (Labsolar 6A) was used to evaluate the photocatalytic performance of the samples. A closed quartz reactor was charged with the photocatalyst (5 mg), acetonitrile (MeCN, 4 mL), triethanolamine (TEOA, 1.5 mL), H_2O (1 mL) and $Ru(bpy)_3Cl_2 \cdot 6H_2O$ (10 mg). Before light irradiation, degassing with CO_2 was performed to remove trace gases inside the reactor. The reaction mixture was illuminated for 4 h with a 300 W Xe light source equipped with a $\lambda > 420$ nm cut-off filter. Every 30 minutes, the content of CO was detected qualitatively and quantitatively by a gas chromatograph (Fuli GC9790ll) equipped with FID.

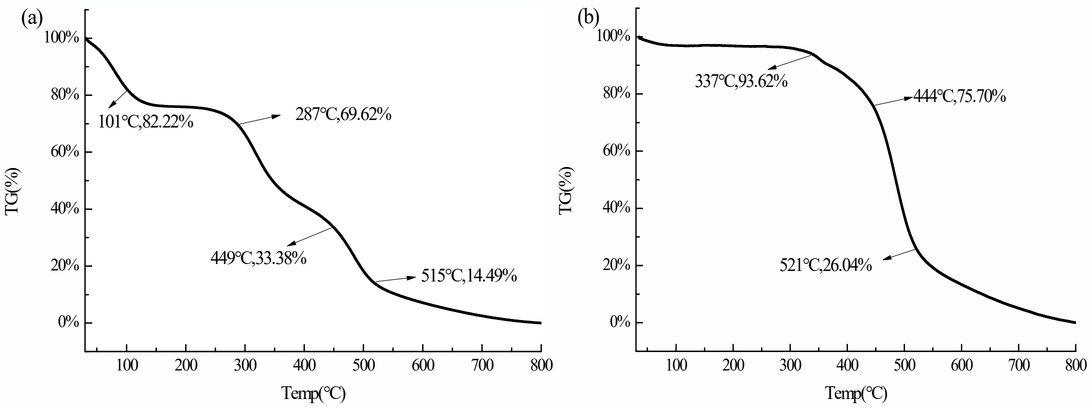

Fig.S3. TG profile of (a) TpPa-SO_3H and (b) TpPa.

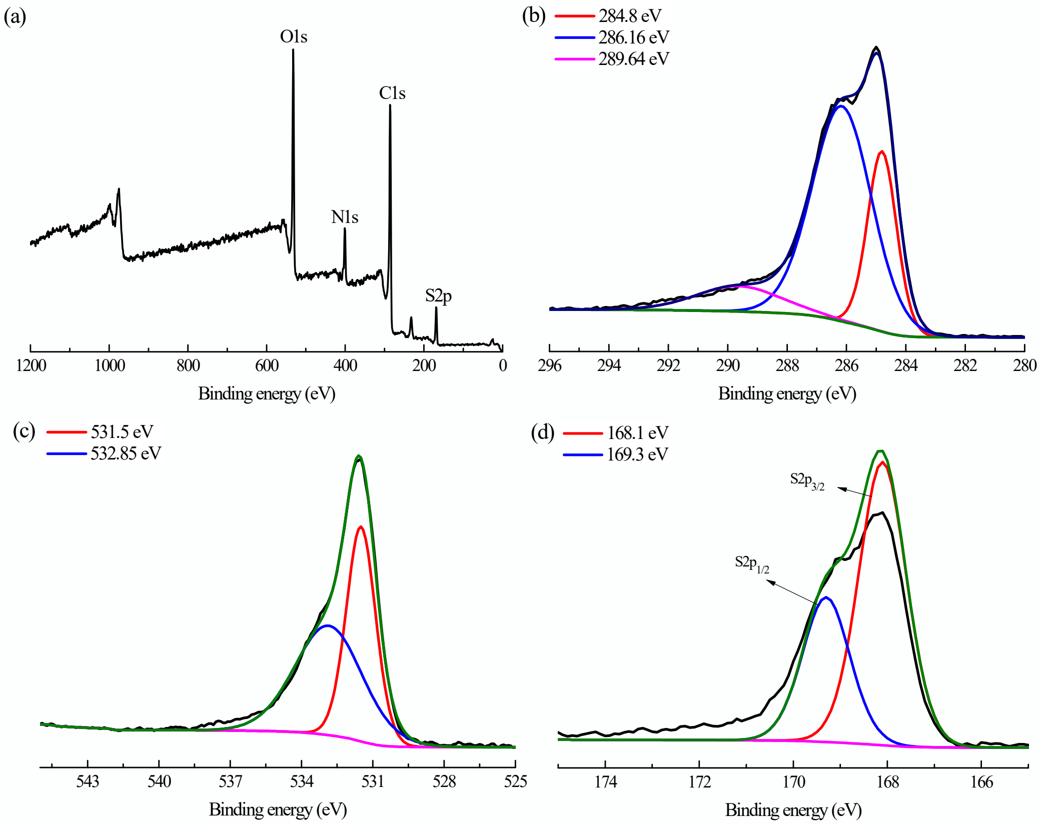

Fig.S4. XPS survey spectra of TpPa-SO_3H (a) full spectrum (b) C1s (c) O1s (d) S2p.

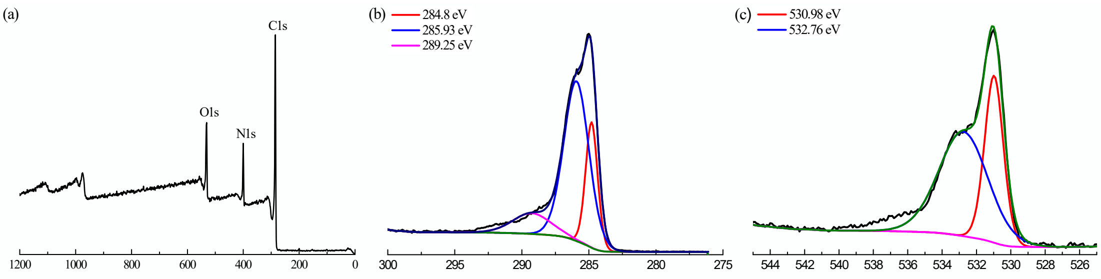

Fig.S5. XPS survey spectra of TpPa (a) full spectrum (b) C1s (c) O1s.

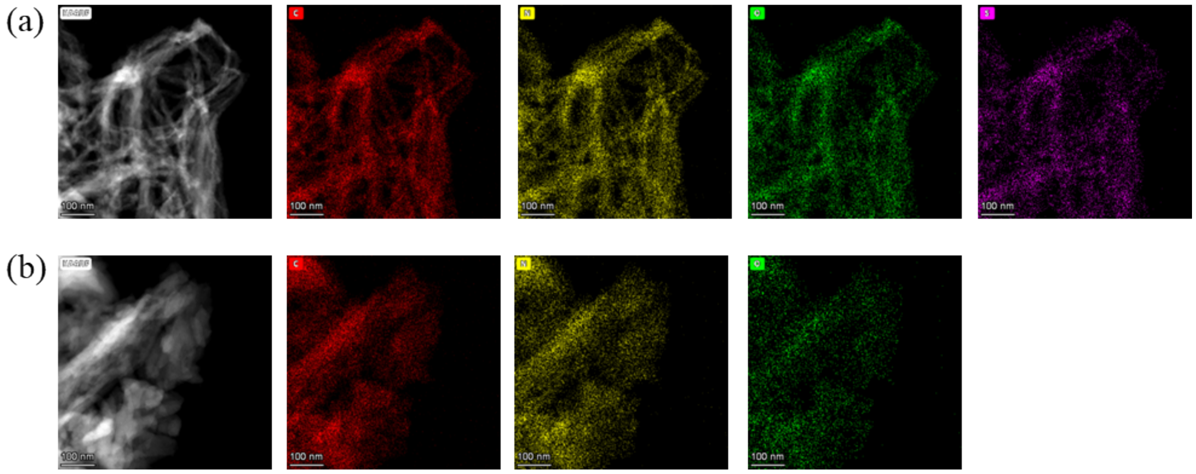

Fig.S6. EDX elemental mapping of (a) TpPa-SO_3H and (b) TpPa

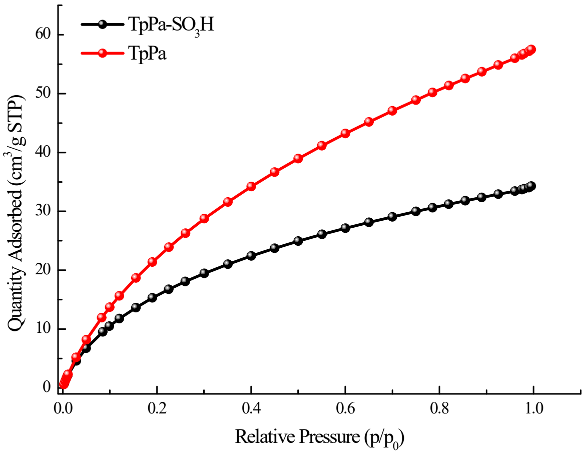

Fig.S7. CO_2 adsorption isotherms of TpPa-SO_3H and TpPa

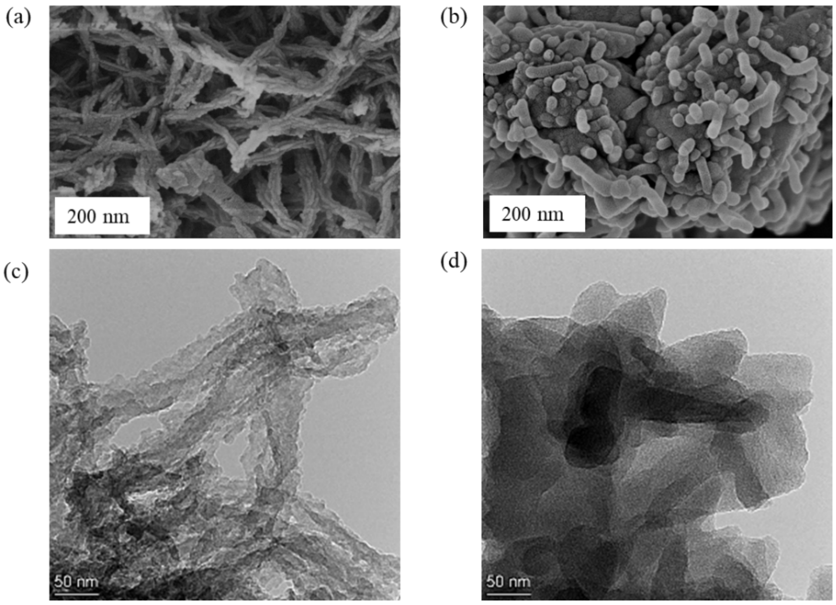

Fig.S8. SEM image of (a) TpPa-SO_3H and (b) TpPa. HRTEM image of (c) TpPa-SO_3H and (d) TpPa.

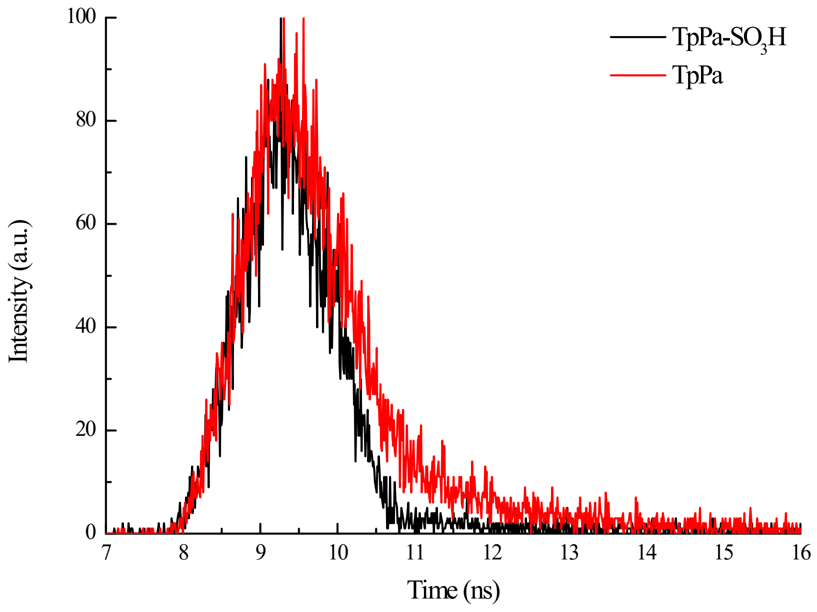

Fig.S9. TRPL spectra of $TpPa-SO_3H$ and TpPa.

Table S1. PL decay parameters (λex = 370 nm, λem = 610 nm).

**Sample**	**τ(ns)**	χ^2
$TpPa-SO_3H$	0.5	1.105
TpPa	0.8	1.017

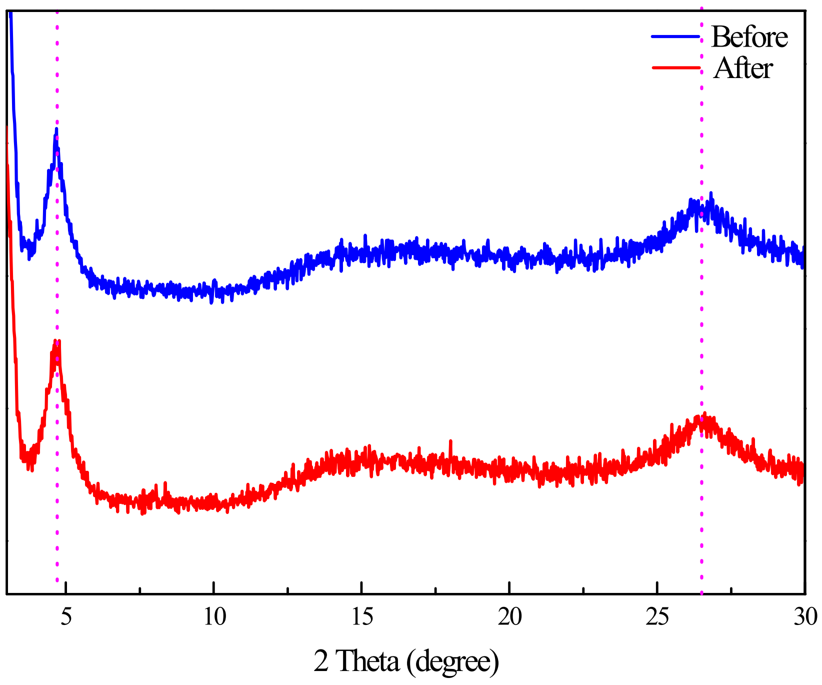

Fig.S10. XRD comparison chart before and after $TpPa-SO_3H$ catalysis.

Table S2. Comparison of performance of various photocatalysts for reducing CO_2

Photocatalyst	Reaction time	Yield	Reference
TpPa-SO_3H	4h	416.61 $\mu mol \cdot g^{-1}$	This Work
TpPa	4h	380.68 $\mu mol \cdot g^{-1}$	This Work
CdSe/ZIF-8	12h	42.317 $\mu mol \cdot g^{-1}$	[3]
g-C_3N_4-RGO-MOF	4h	383.79 $\mu mol \cdot g^{-1}$	[4]
Bi_2S_3 QDs/g-C_3N_4	8h	54.74 $\mu mol \cdot g^{-1}$	[5]
PMA@NH_2-MIL-68(Rh)	10h	387.28 $\mu mol \cdot g^{-1}$	[6]
Sm-TiO_2 nanoparticles	20h	55.47 $\mu mol \cdot g^{-1}$	[7]

Notes and references

1 K. Jeong, S. Park, G. Y. Jung, S. H. Kim, Y. H. Lee, S. K. Kwak and S.Y. Lee, *Journal of the American Chemical Society*, 2019, **141**, 5880-5885.

2 H. Yang, H. Wu, Z. Xu, B. Mu, Z. Lin, X. Cheng, G. Liu, F. Pan, X. Cao and Z. Jiang, *Journal of Membrane Science,* 2018, **561**, 79-88.

3 H. J. Peng, P. Q. Zheng, H. Y. Chao, L. Jiang and Z. P. Qiao, *RSC Advances*, 2019, **10**, 551-555.

4 R. R. Ikreedeegh and M. Tahir, *Journal of Environmental Chemical Engineering*, 2021, **9**, 105600

5 R. T. Guo, X. Y. Liu, H. Qin, Z. Y. Wang and J. W. Gu, *Applied Surface Science,* 2019, **500**, 144059.

6 Y. Shan, D. Liu, C. Xu, P. Zhan, H. Wang, J. Wang, R. He and W. Wang, *New Journal of Chemistry*, 2021, **45**, 7344-7352.

7 H. Peng, R. Guo and H. Lin, *Journal of Rare Earths*, 2020, **38**, 1297-1304.
